# Influenza and Pneumonia

**Published:** 1900-01-20

**Authors:** 


### Influenza and Pneumonia.

The remarkable outburst of influenza which has spread over certain parts of England during the past few weeks, and the extent to which the death-rate has thereby been raised—largely in consequence of the intercurrence of inflammation of the lung—renders the subject of pneumonia one of immediate interest to all practitioners. Nor can it be said that a disease like pneumonia is ever lacking in interest and importance, even at ordinary times, seeing that, according to Dr. Hector Mackenzie,[1] in a population of 30 millions at least 220,830 persons are affected by it in the course of a year, 31,950 of these persons dying from its effects. Clinically, and in its classical form, pneumonia is one of the most distinct and definitely marked of the acute febrile diseases, with sudden onset, high fever, sharply drawn symptoms, and rapid defervescence. Pathologically, however, in addition to attacking the healthy and the strong in the sthenic form, pneumonia often seems to be a mode rather than a prime cause of death. This is markedly the case in influenza, in which, especially in the old, pneumonia is one of the most fatal of complications.

Regarded from another point of view, however, pneumonia is a purely microbial affair, an infection by the pneumococcus; and if one had space to enlarge upon this aspect of the question, doubtless many lessons might be learned as to the relation between predisposing and exciting causes in the production of disease, not merely in regard to susceptibility and immunity, but in reference to the varied character or type of malady which may be set up by one and the same infection. Suffice it here to say that while those living under the best hygienic conditions, and not mixing too much with their fellows, no doubt escape many risks of becoming affected with pneumonia, the great majority, who live the ordinary gregarious life in which mankind delights, carry about with them in one or other of their secretions a certain charge of the infection—harmless under ordinary circumstances, but sufficient to set up the disease under certain conditions, such as those caused by a chill, an exhausting illness, and a weakened circulation, or the mere enfeeblement of old age.

Then only, as a rule, does the enemy attack us. But in exceptional circumstances, related probably with conditions of the dwelling, the pneumococcus attains to a far greater degree of virulence, and then we have to do with that rare but well-recognised condition, "infectious pneumonia." What are the conditions which lead to this increased infectivity are not yet known, but they almost certainly have to do with dirt and overcrowding. Dr. Newsholme[1] gives the following, among other examples, of the infectiousness which sometimes attends cases which in their symptoms do not appear to differ from ordinary cases of pneumonia: "A clergyman was attended by a nurse for acute pneumonia; in about seven days she contracted the same disease. The clergyman's sister, who then took the place of the nurse, was in her turn seized with pneumonia. A brother of the clergyman next came to help, and he in his turn was next laid up with the same disease." Again, "A shopkeeper's child was convalescing from pneumonia. About the same time (December 10th) the servant-boy began with pneumonia. He was taken to the hospital, a second boy taking his place, wearing the same clothes and sleeping in the same bed as his predecessor. Two days later he failed with pneumonia. A third boy was engaged on December 18th; he slept for two nights with the second boy, and 30 hours later failed with pneumonia." Dr. Newsholme adds that all the accounts of epidemic pneumonia agree in associating its occurrence with conditions of overcrowding, and especially with defective conditions of house sanitation involving privy or drain effluvia.

Now there are degrees and gradations in all things, and one can hardly avoid the conclusion that between the extreme cases—the apparently healthy man struck down with pneumonia after prolonged exposure on the one hand, and on the other the case in which by dint, apparently, of sheer virulence the disease spreads from patient to patient—there are many steps, and that often it is to the surroundings amid which people dwell rather than to accidental hardship or exposure that they owe the pneumonia from which they die. This is markedly the case in regard to the lobular pneumonia of children, which is so largely responsible for the heavy fatality of measles and whooping cough among the poor. Often this is a mixed infection,[2] the secondary microbes involved in the process being found in the overcrowded rooms, and being capable of being conveyed from person to person by drinking vessels in common use and in other ways. "Thus is to be explained the extreme frequency and virulence of broncho-pneumonia among children suffering from measles and whooping-cough nursed in overcrowded tenements."[3]

Everywhere we meet with the same lesson—that of the advantage of the cleanly life. Anyone may become affected with pneumonia; of that we must all take our chance. But the gravity of the attack will probably depend upon the extent to which the virulence of the infection and our own susceptibility have been aggravated by insanitary surroundings, and upon the extent to which the disease is a sequel to some exhausting malady, such as influenza. The first condition is a matter which depends upon ourselves to a large, the latter to a less degree. But even in regard to the latter, it is worth bearing in mind that most people could do far more than they do to protect their aged relatives from the infection of influenza; and in view of the tendency which that disease shows at the present time to become complicated with pneumonia—an almost fatal complication—we think much more care should be exercised than is commonly done in this respect.

[1] Practitioner, January, 1900. [2] Pye-Smith, in "Allbutt's System of Medicine." [3] Dawson Williams, "Medical Diseases of Infancy and Childhood."

